# Natural Occurring Silks and Their Analogues as Materials for Nerve Conduits

**DOI:** 10.3390/ijms17101754

**Published:** 2016-10-20

**Authors:** Christine Radtke

**Affiliations:** Department of Plastic and Reconstructive Surgery, Medical University of Vienna, Währinger Gürtel 18-20, 1090 Vienna, Austria; christine.radtke@meduniwien.ac.at; Tel.: +43-01-404-006-9860; Fax: +43-01-404-006-9880

**Keywords:** Schwann cells, axonal regeneration, remyelination, nerve defect injury, spider genus *Nephila clavipes*

## Abstract

Spider silk and its synthetic derivatives have a light weight in combination with good strength and elasticity. Their high cytocompatibility and low immunogenicity make them well suited for biomaterial products such as nerve conduits. Silk proteins slowly degrade enzymatically in vivo, thus allowing for an initial therapeutic effect such as in nerve scaffolding to facilitate endogenous repair processes, and then are removed. Silks are biopolymers naturally produced by many species of arthropods including spiders, caterpillars and mites. The silk fibers are secreted by the labial gland of the larvae of some orders of Holometabola (insects with pupa) or the spinnerets of spiders. The majority of studies using silks for biomedical applications use materials from silkworms or spiders, mostly of the genus *Nephila clavipes*. Silk is one of the most promising biomaterials with effects not only in nerve regeneration, but in a number of regenerative applications. The development of silks for human biomedical applications is of high scientific and clinical interest. Biomaterials in use for biomedical applications have to meet a number of requirements such as biocompatibility and elicitation of no more than a minor inflammatory response, biodegradability in a reasonable time and specific structural properties. Here we present the current status in the field of silk-based conduit development for nerve repair and discuss current advances with regard to potential clinical transfer of an implantable nerve conduit for enhancement of nerve regeneration.

## 1. Introduction

Silks are thin structural protein fibers produced by arthropods including almost all insect taxa, but spiders are the dominant species for silk production [1,2,3]. Spider silks serve many biological purposes such as webs to catch other animals and are produced by several glands (colleterial, salivary, dermal glands, Malpighian tubes) at different stages of arthropod development [4]. They are spun into fibroin fibers for webs, egg cases and cocoons, but are also used as fibrous sheets and glues.

Spiders are unique among silk-producing arthropods in that they produce silk throughout their lifetimes. The orb-spinning spiders produce different types of silk in specialized glands adaptive to specific purposes and availability of nutrients [4]. Silk glands are found in the abdomen of spiders and are connected to the spinnerets by ducts. As the fibers exit the spinnerets they polymerize from stored silk monomers in a pH-regulated self-assembly process (reviewed by [5]). The conformational changes associated with the spinning process lead to the formation of pseudocrystalline regions of antiparallel beta-sheets interspersed with elastic amorphous segments which are responsible for the remarkable mechanical properties of the fibers [6].

Silkworm–derived silks have been used for the production of textiles and for surgical sutures [1,7]. They were domesticated more than 5000 years ago and are kept in more than 1000 inbred lines worldwide in so-called sericulture [8]. Predatory spiders cannot be kept in intensive husbandry as silkworms can, and their silks have only been used for specialized purposes. In ancient times cobwebs were used for hemostasis in bleeding wounds [9] and some societies used spider silk for fishing lines, an application which is still practiced in the Solomon Islands [5]. The industrial production of spider silk must take into account that it is harvested in much lower quantities per animal than the silk of silkworms. Additionally, there is considerable variability in production depending not only the species of spider, but on individual spiders and the harvesting process [10].

Structurally, spider dragline silk consists of long repetitive stretches containing four basic amino acid motifs (An, GA, GGX, and GPGXn) enclosed by non-repetitive C- and N-terminal regions [11]. There is high variability among spider silk-encoding genes which depends on allelic variants. Multiple intra-species and even intra-individual alleles exist, but there is negative selection pressure on the protein size, typically keeping them in a range between 200 and 350 kDa [12]. *Nephila clavipes* dragline silk which we use for the construction of nerve conduits is mainly composed of two proteins: MaSp1 and MaSp2 which are uniformly distributed in the fiber [13]. The spidroin’s structural composition allows them to incorporate water into their amorphous regions, inducing recoiling of the fibers and reversible shrinking upon contact with water or in high humidity [14].

In this review we first describe the current standard use of nerve grafts and the development of bioengineered nerve conduits for nerve repair. We then discuss the development of spider silk-based nerve conduits and their application for in vivo nerve repair, thus providing a preclinical basis for their potential use as a nerve conduit for clinical nerve repair.

## 2. Peripheral Nerve Repair by Nerve Grafting or Conduit Implantation

Nerve damage or loss from severe trauma can result in complete functional deficit of the injured extremity. A special challenge with regard to nerve injury is the repair of long distance nerve defects referred to as a nerve substance defect injury. Long distance nerve defects typically entail greater than 4 cm of nerve loss [15] and the development of effective surgical treatments for substance defect injury is of considerable clinical interest. The standard procedure for nerve repair is surgical intervention with nerve suturing for simple nerve transections (direct coaptation) or autologous nerve transplantation for longer nerve defects [16,17]. However, the results are often disappointing and methods to improve functional outcome with grafting procedures are necessary [18]. Sensory nerves (e.g., the sural nerve or medial cutaneous nerve) derived from the patient are harvested for nerve grafting. Although no motor loss results from surgical harvesting of the donor sensory nerves, there is inevitable donor morbidity including sensory loss and possible neuroma formation after donor nerve removal, which may lead to chronic neuropathic pain. While sensory loss may occur from nerve harvest, grafting these nerves for the reinnervation of hand muscles, for example, could potentially result in the considerable recovery of essential motor function. Major limitations of nerve graft procedures include the limited amount of available donor nerve and that grafts greater than about 4–6 cm show poor regeneration and functional recovery [15,19,20].

Allograft or xenograft transplantations are being discussed as an alternative to autologous nerve transplantation [21], but a major concern is the potential side effects of immunosuppression which is required for this approach. Autologous nerve grafts have limitations both in terms of the length and diameter of nerve that can obtained. Moreover, there is the potential donor for site morbidity. Much research is currently underway to develop artificial nerve conduits which may serve as guiding channels for regenerating axons thus reducing the need for donor tissue [22,23]. Tubular nerve guidance channels have the transected nerve stumps inserted into them on both sides; the proximal nerve stump can issue regenerating axons that grow though the guidance channel into the distal nerve stump and to target tissue. Natural biological tissue such as autologous veins, arteries or skeletal muscle can be used as materials for conduit construction. Synthetic materials including silicone and chitosan [24,25] or biodegradable polymers such as poly l-lactic acid (PLLA), polyglycolic acid (PGA), poly-3-hydroxybutyrate (PHB) and their copolymers or derivatives [26,27,28] are being actively researched for conduit construction as alternatives for biological tissues. A variety of nerve tubes have been marketed [23,29,30]. Following application of a collagen-based nerve tube that is available for treating short nerve defects, a neuroma formation was reported after implantation into a 2 cm defect [31], suggesting that existing collagen-based guides are a possible choice for long nerve defect reconstruction [32].

In general, commercial nerve tubes are only approved for up to 2 cm defects and are not as effective as autologous nerve grafts, thus emphasizing the need for development of bioengineered scaffolds that could be used for longer nerve defects. Experimental studies demonstrated that tubular repair of short 3–5 mm nerve gap tubes yielded functional recovery at least as good as routine microsurgical repair in the short term [33,34]. In the long term, these grafts may become harmful by virtue of toxicity or tendency to constrict the nerve [35,36]. It should be pointed out that the relative efficacy between nerve grafts and hollow tube repair is difficult to evaluate because different model systems have been used and are not easily compared [37].

Success of tubular scaffold implantation requires that the conduit material be properly selected so as not to be toxic or induce an immunological response [38]. A biodegradable conduit is important because permanent tubes may induce a foreign body reaction and inhibit nerve growth by scar tissue formation. If, during the conduit degradation, time-dependent swelling of the tube occurs, this could lead to a reduction of the tube diameter and to nerve compression. If the conduit wall is too thick, degradation will be slow, thus increasing the time of possible foreign body reaction [38,39]. Conduits with thin walls have the risk of collapse. A biodegradable nerve guide with an internal diameter of 1.5 mm and a wall thickness of about 0.3 mm was found to be optimal for peripheral nerve regeneration in rodents [30,40].

In nerve repair, axons must bridge a nerve gap in a reasonable period of time to reach the degenerating distal nerve segment, which is trophic for nerve regeneration, before more complete degeneration of the distal segment with axon-impeding scarring occurs. The distal degenerating nerve stump provides guidance for regenerating axons through the tubular basement membrane, trophic support from Schwann cells dissociated from degenerating axons and immune cell infiltration which supports nerve regeneration [41]. Schwann cells produce both nerve growth factor (NGF) and NGF receptor and other neurotrophins in the degenerating nerve segment which supports regenerating axons [42]. However, excessive NGF exposure can be detrimental by leading to retrograde changes in the expression of ion channels of sensory neurons, resulting in hyperexcitability and extreme pain [43]. Thus, care must be taken in consideration of trophic factor additions to artificial conduits.

Simple nerve tubes have been modified to incorporate a supporting structure or substance to stabilize the tube [44]. These include collagen- or laminin-containing gels which can be combined with growth factors or cells such as Schwann cells to enhance regeneration [26]. Another approach for optimization of nerve conduits is the production of nerve guidance channels to provide chemotactic and topological signaling and the use of internal-oriented matrices/fibers with possible release of neurotrophic factors [44]. A number of important advances in the laboratory are underway with regard to building conduits with multiple components including nanotechnology, slow release of growth factors [45], and seeding with Schwann cells [46] or stem cells [47]; however, these have not yet reached clinical applicability.

## 3. Natural Silk and Silk Analogue-Based Guidance Conduits for Peripheral Nerve Regeneration

A number of investigators are currently working with silk as a material for tissue engineering. Different forms of silk including natural occurring silk, silk fibroins harvested from natural sources or artificial sources including recombinant silk are being explored for their potential biomedical application. Natural occurring silks, e.g., spider silk or cocoon silk from the silkworm *Bombyx mori*, have been shown to be advantageous for cell attachment. Spider silk, when used for long distance nerve regeneration with long implantation times and slow degradation [48], did not induce an inflammatory foreign body response or result in non-physiological pH changes, which could inhibit tissue regeneration. The use of silk fibroin proteins, however, is a different approach to the use of spider silk as a processed material is used instead of native biomaterial [49]. The processing allows for technical adaption such as tube formation by gel spinning or combinations with other materials. Studies have demonstrated, as described below, that silk fibers are compatible with neuronal cells, they are biodegradable and they can be used as part of the nerve conduit for peripheral nerve repair.

### 3.1. Silk-Neuronal Compatibility

Silk protein polymers are well established for biocompatibility and have, for centuries, been used for wound healing and surgical sutures [1,9]. Recent interest in silks has led to a number of recent studies related to safety and efficacy. Potential cytotoxicity of silk fibers to neurons was tested on dorsal root ganglia (DRG) neurons from neonatal rats cultured with short silk fibroin fibers [49]. Silk fibroin fibers were obtained by boiling *Bombyx mori* cocoons in aqueous 0.5% Na_2_CO_3_. The cells leaving the DRG adhered to and migrated on the substrate. DRG neurite outgrowth (seven days in culture) was observed along the fibroin fibers, and Schwann cells identified by immunocytochemistry associated with the neuritis, forming single layers and multilayers. An indirect test for cytotoxicity was performed by culturing Schwann cells in fibroin-conditioned media. No significant differences in cell phenotype or proliferation were observed, confirming that the silk fibroin extract had no deleterious effect on Schwann cells in culture [49]. In a subsequent study, the cytocompatibility of silk fibroin with embryonic rat hippocampal neurons isolated from comparable experimental conditions was assessed. Protein levels of phospho-PI3K and phosphor-PKB/Akt were measured in relation to the total protein content as key elements of the PI3K survival pathway. Neuronal networks were observed after seven days of culture and again no harmful effects on cell viability and phenotype were observed in direct and indirect approaches. Thus, they conclude that silk fibroin is nontoxic to neurons and glial cells and should be further studied for nerve guidance or drug delivery in central nervous system injuries or diseases [50].

Human mesenchymal stem cells (hMSCs) might support nerve regeneration due to their production of trophic factors which could be neural-protective and stimulate regeneration [51]. Therapeutic approaches involving the regeneration properties of mesenchymal stem cells are a promising development in neuroregenerative therapies. Garcia-Fuentes et al. (2009) investigated the early inflammatory responses of hMSCs on hexafluoro-2-propanol–extracted silk films [52]. The scaffolds either contained randomly dispersed or aligned fibers; the randomly patterned scaffolds were either coated with fibronectin or left uncoated. The seeded human MSCs reacted to the different matrices: fibronectin coating enhanced the adherence of the cells and they migrated along the fibers [52].

While a number of studies indicate the cytocompatibility of substrates derived from *Bombyx mori* cocoons, much less is known about the tolerance of spider silk by neurons. Human Schwann cells isolated from peripheral nerves and olfactory ensheathing cells adhered to native spider silk fibers with high viability (48 h), suggesting no toxic or harmful effects on the cells. Silk fibers were collected directly from the major ampullate gland of adult female *Nephila clavipes* spiders and were used in their native form [48,53]. Subsequently, a nerve conduit was developed based on these data, consisting of spider silk fibers aligned within a decellularized vein. When supplemented with Schwann cells, they aligned along the fibers [48,53] and migrated into the conduit after in vivo application [48].

It was demonstrated that natural dragline silk incorporated into isogenic or decellularized veins and sutured into an injured peripheral nerve promoted peripheral nerve regeneration [48,54]. Axon regrowth and remyelination were achieved by replacing a 2 cm segment of sciatic nerve in rat and a 6 cm segment of tibial nerve in adult sheep [47,54]. The results obtained were comparable to autologous nerve transplantation which is the current standard in clinical practice.

### 3.2. Biodegradability of Silk

Nerve conduit biodegradability is desirable to avoid a second operation for implant removal or potential long-term adverse effects of the implanted material [38,55]. To serve as axon guides, nerve conduits should degrade at controllable rates, allowing sufficient time for axonal regeneration but for later degradation [38,56]. As a result of their compact and crystalline structure, silks in general degrade slowly. These proteinaceous fibers can be degraded by proteolytic enzymes including chymotrypsin, actinase and carboxylase [7]. Degradation rates of regenerated silk are typically faster, likely the result of changes in conformation and molecular weight by the dissolving process [57].

Short-term observations on silk degradation have been made in vitro. Scaffolds prepared from *Bombyx mori* fibroin and silk carbon nanotube controls became porous when they came into contact with human embryonic stem cells over an in vitro incubation period of seven days [58]. Degradation of micropatterned films cast from dissolved fibroin solution was also observed in an in vitro hydrated environment over two weeks [59].

Most in vivo degradation was reported in studies with long-term follow-up. In a study which implanted a fibroin-based nerve conduit in a rat model, degradation of their implants was documented after six months. They hypothesized that the preceding treatment of the fibroin by dissolving it in a highly concentrated calcium chloride solution, which changed the molecular weight and protein conformation, allowed for greater accessibility of proteases [49,59]. In another study a nerve conduit made of regenerated and electrospun *Bombyx mori* fibroin implanted into the facial nerve of rats was fully degraded and replaced by a tissue with nerve-like appearance after three months [60].

Complete degradation of spider silk fibers was reported after six months subsequent to implementation in a nerve conduit used for a sciatic nerve replacement over a gap of 20 mm [54]. In a subsequent study, when the same nerve conduit design was used to bridge a defect of 60 mm in the tibial nerve of sheep, no traces of spider silk could be observed after 10 months [48]. Thus, these studies indicate that the silk material used in nerve conduits biodegrades over time.

## 4. The Development of a Spider Silk/Acellularized Vein-Based Nerve Conduit

The use of native spider silk was considered because of its low immunogenicity and degradation properties, but general use was not initially considered due to the potential difficulty of harvesting silk directly from the spider. Additionally, the cannibalistic nature of the spiders presented mass production problems. However, these issues have been resolved and relatively large amounts of spider silk can be harvested. The use of native spider silk has been successfully established as a biomaterial for nerve regeneration and other biomedical applications [47,48,61,62,63,64,65,66,67,68]. As a first step for the development of a spider silk-based nerve conduit, a breeding station for spiders of the genera *Nephila clavipes* was build and processing parameters of native spider silk were established. Procedures were developed to harvest the native dragline silk directly from the adult female spiders in relatively large quantities [61].

*Nephila clavipes* spider silk was seeded with NIH-3T3 murine fibroblasts. The cells attached to the spider silk and grew over an incubation period of 20 days without observable toxic effects [66]. This demonstrated that the harvested spider silk supports cell growth and adhesion of fibroblasts and induces directed growth.

Moreover, the combination of human model neurons (Ntera neurons) and spider silk showed that within two hours after seeding, there was attachment of the neurons to the silk [67]. Aggregates of cell bodies and neurites crossing the gap between individual silk fibers occurred within several hours in culture. Silk-attached neurons gave rise to neurites which extended along the silk fibers, using them as guidance structures and an outgrowth substrate. The silk fiber-attached neurons also sent neurites over a 50 mm gap between single silk fibers and connected to an adjacent silk fiber. Neurites attached to the silk fiber and bifurcated, formed small hooks, and grew spirally around the fiber.

With time (36 h), additional neurons were recruited to the silk fibers with their cell bodies oriented along the fibers. The spider silk–attached clusters of neurons increased in size for five days. The neurons comprising the clusters extended their neurites in the surrounding area and stayed in contact with neurons not part of the ganglion-like aggregates. After three to four weeks of culturing human neurons on single spider silk fibers, the ganglion-like structures enlarged and formed thick bundles of neurites along the silk fibers which established contact with other ganglion-like structures. Thus, the manufactured spider silk fibers provided for aggregation of neurons in vitro and for linear guidance of neurites which is an important consideration for their use in a nerve conduit. For in vivo applications to make spider silk fibers usable as guiding structures for nerve regeneration of nerve defects a construct was developed, in which silk fibers were integrated into either decellularized or isogenic veins providing thus an internal framework [48,54].

A simple and novel artificial nerve conduit which consists of unique spider silk fibers fed into an acellularized vein was developed to support nerve regeneration [48,54]. The acellularized vein serves as a tubular nerve guide that can be sutured to the proximal and distal ends of the transected nerve, and the fine spider silk fibers serve as an internal guidance material for individual axonal regeneration [48,54]. Spider dragline silk from the spider *Nephila clavipes* has novel characteristics regarding nerve regeneration guidance and spider dragline silk can be easily harvested at long lengths (up to 100 m). The tensile strength of the spider silk is up to 4.8 GPa; comparable to Kevlar [68], it is lightweight (1.3 g/cm^3^) [69] and it is thermally stable up to about 220 °C [70]. The constructs can be used cell-free or prepared with isogenic Schwann cells (or other cells); veins without spider silk and isogenic grafts were used as negative and positive controls, respectively. These constructs were then tested as a conduit for nerve repair in both small and large animal models.

## 5. Application of the Spider Silk Conduit for Nerve Repair in Small and Large Animal Models

Surgical implantation of nerve constructs composed of acellularized vein grafts filled with linearly aligned spider silk fibers serving as a nerve guidance material displayed remarkable nerve regeneration in both rodents and sheep. In these in vivo settings, the spider silk showed nearly complete biodegradability over time without observable immunological or toxic effects [47,48,54]. The acellularized vein not only provides a structural encasement for the silk fibers, but it also provides a surface similar to the epineurium to allow nerve suture repair of the graft. The rate of growth of regenerating nerve fibers and the time course of biodegradation of the spider silk was determined.

A large animal (sheep) was selected for testing the spider silk grafts because it reproduces specific pathologic processes which take place in human peripheral nerve injury. Advantages of using sheep include that: (1) long nerve defects can be created and (2) sheep nerves are spatially similar to those in humans and can simulate those of humans. While rodents are important in initial testing of nerve constructs, they present issues of scale which can be addressed in large animal studies.

**Figure 1:**
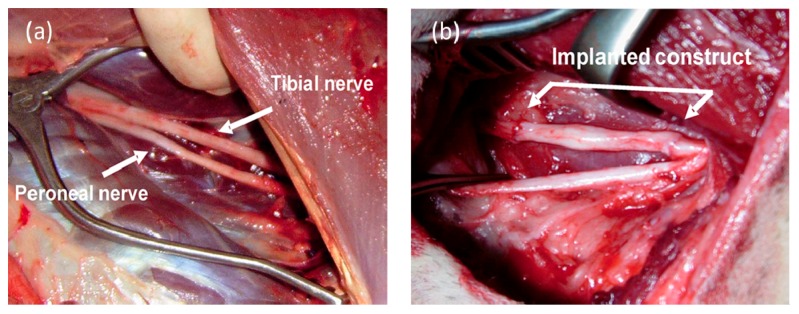
Nerve defect induction in sheep peripheral nerve and spider silk–based construct implantation. (**a**) Tibial and peroneal nerves (arrow head) in the sheep; (**b**) Six cm nerve defect and bridging of nerve defect with vein/spider silk construct sutured between proximal and distal nerve stumps of the nerve defect in an adult sheep. From Radtke et al., 2011 [48].

A decellularized xenogenic vein with inserted spider silk fibers was tested in a critical size defect of 6 cm in the tibial nerve of sheep against an autologous nerve graft as a positive control (Figure 1).

Electromyography and electroneurography were performed after 6 and 10 months and improved electrophysiological function was observed at both time points. No adverse effects were observed and histological analysis indicated no signs of foreign body reaction or an immune response as described in Radtke et al. [48].

**Figure 2:**
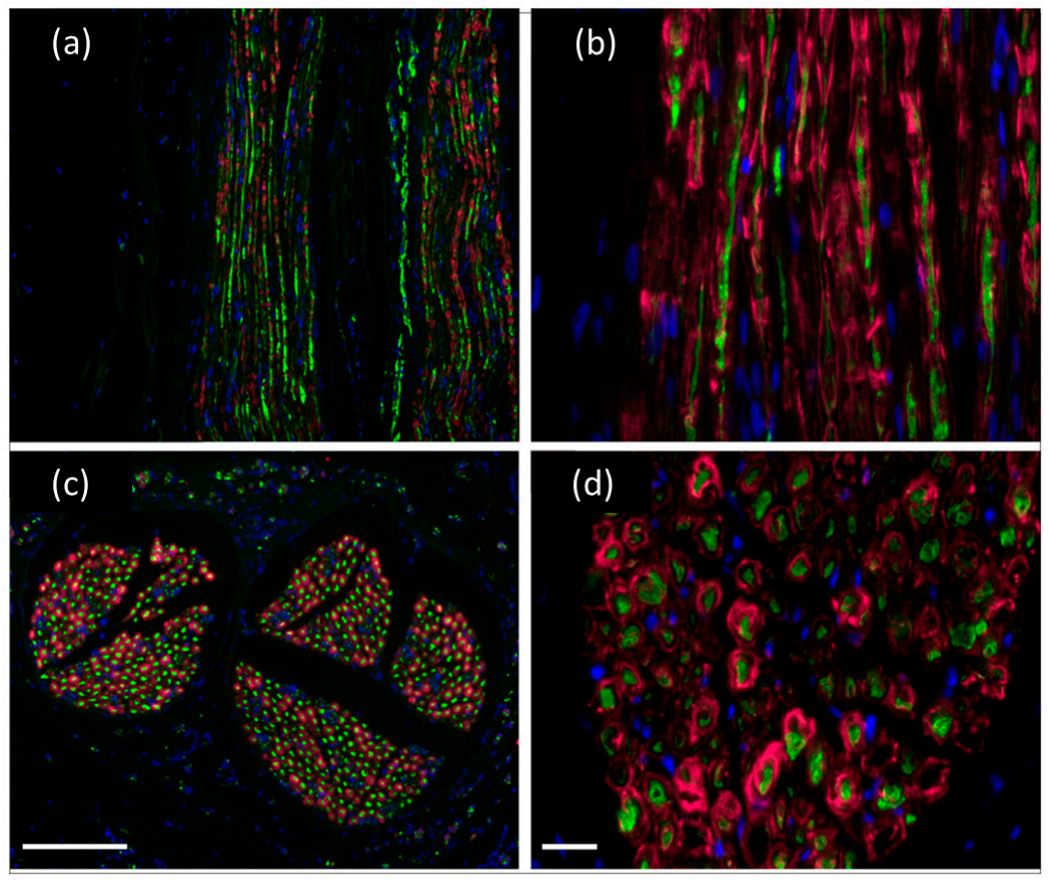
Regenerated nerve fibers following spider silk construct implantation: (**a**,**b**) Longitudinal section through the construct showing regenerated nerve fibers and Schwann cells in the construct identified with neurofilament (NF, green) and S100 immunostaining (red), respectively, demonstrating that axons regenerated and endogenous Schwann cells migrated within the construct); (**c**) Cross-sections of regenerated nerve after implantation of spider silk construct showed immunopositive staining for S100. Co-association of Schwann cells indicated remyelination of the regenerated nerve fibers in the construct (**c**,**d**). Nuclear staining with DAPI (blue). Scale bar in (**c**) = 50 µm and pertains to (**a**,**c**); scale bar in d = 8 µm and pertains to (**b**,**d**). From Radtke et al., 2011 [48].

Axonal regeneration within and through the spider silk–based construct was demonstrated by positive neurofilament staining (axons) and S-100 positive staining demonstrated that endogenous Schwann cells migrated into the nerve constructs (Figure 2). A large number of axons grew through the constructs, leading to extensive morphological and functional regeneration of the nerve. Schwann cells can serve as nerve guides and produce important trophic factors to stimulate axonal regeneration. There was extensive endogenous Schwann cell migration into the acellularized spider silk construct (Figure 2), indicating that both regenerating axons and their supporting glial cells associate with the spider silk fibers [48]. The endogenous Schwann cells migrated along the spider silk fibers associated with the regenerated axons and formed myelin (Figure 2d) which is essential for restoration of rapid and secure impulse conduction [48]. The sodium channel subtype, Nav1.6, is the primary sodium channel at normal nodes, and it was present at the nodes of regenerated axons in the construct.

Interestingly, no significant difference in regeneration was observed between the spider silk constructs and autologous tissue grafts [48]. This is important because autologous nerve grafts are the current standard practice in reconstructive nerve surgery. These data suggest that spider silk constructs may be a substitute material obviating the need for autologous nerve grafts which present a degree of morbidity [71]. Thus, the demonstrated efficiency of these novel nerve constructs has important clinical implications for nerve repair.

## 6. Conclusions

Spider (genus *Nephila*) silk is biocompatible with neural tissue and is biodegradable over time. Neurons and glial cells adhere and migrate on the silk fibers both in vitro and in vivo. Acellularized xenogenic veins supplemented with spider silk fibers microsutured into a critical size defect of 6 cm in the tibial nerve in sheep support axonal regeneration and improved electrophysiological and motor function. This is evidenced by a large number of axons and endogenous Schwann cells growing through the constructs, leading to substantial morphological and functional regeneration of the nerve through the conduit, similar to that of the autologous nerve. This experimental model demonstrating that silks can lead to nerve repair in a large animal model is a significant step in developing a silk-based bioengineered nerve construct that can substitute for autologous nerve grafts.

## Abbreviations

DRG	Dorsal root ganglia
hMSCs	Human mesenchymal stem cells
NGF	nerve growth factor
NIH-3T3	murine fibroblasts
Ntera	Human model neurons
PGA	polyglycolic acid
PHB	poly-3-hydroxybutyrate
PLLA	poly l-lactic acid Three letter acronym
